# Supplementary material for: IL-10 Producing B Cells Protect against LPS-Induced Murine Preterm Birth by Promoting PD1- and ICOS-Expressing T Cells
Source: Cells. 2022 Aug 29;11(17):2690. doi: 10.3390/cells11172690 (PMC9454497; doi:10.3390/cells11172690)
Supplement: Supplementary file 1 [file cells-11-02690-s001.zip › cells-1838718-supplementary.pdf]

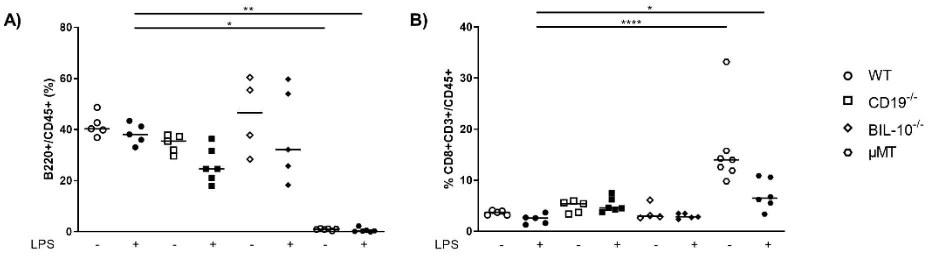

Figure S1: Immune cells in the peritoneal cavity. In the peritoneal cavity, the percentages of B cells (A), CD8+ T cells (B) were measured 24 h after PBS or LPS injection in WT, CD19-/-, BIL-10-/- and μMT dams. N=4-6 mice/group; * $p < 0.05$, ** $p < 0.01$, **** $p < 0.0001$.

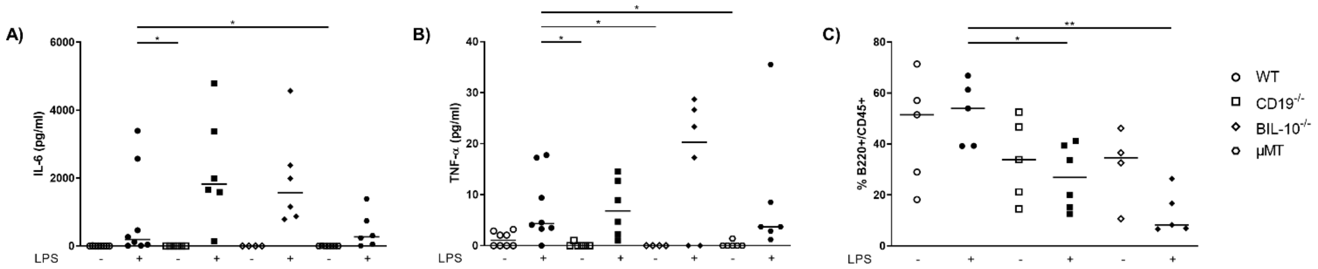

Figure S2: Cytokines and B cell frequencies in blood. Serum level of IL-6 (A) and TNF-α (B) and the frequency of B220+ B cells in blood (C) were determined by flow cytometry. N=4-6 mice/group; * $p < 0.05$, ** $p < 0.01$.

Table S1: Antibodies for flow cytometry staining used in this study

Antigen	Conjugation	Isotype	Clone	Cat. No.	Manufacturer
			Extracellular		
B220	BV605	Rat IgG2a, κ	RA3-6B2	563708	BD Biosciences
CD1d	PerCP-Cy5.5	Rat IgG2b, κ	1B1	562713	BD Biosciences
CD4	FITC	Rat IgG2a, κ	RM4-5	553046	BD Biosciences
CD5	BV421	Rat IgG2a, κ	53-7.3	562739	BD Biosciences
CD8a	APC-Cy7	Rat IgG2a, κ	53-6.7	557654	BD Biosciences
CD11b	BV711	Rat IgG2b, κ	M1/70	563168	BD Biosciences
CD11c	PE-CF594	Hamster IgG1, λ	HL3	562454	BD Biosciences
CD21/35	PE-Cy7	Rat IgG2a, λ	eBio8D9	25-0211-82	eBioscience
CD22.2	BV711	Rat IgG1, κ	Cy34.1	740682	BD Biosciences
CD23	FITC	Rat IgG2a, κ	B3B4	564637	BD Biosciences
CD24	BV421	Rat IgG2b, κ	M1/69	562563	BD Biosciences
CD25	APC	Rat IgG2b, κ	3C7	558643	BD Biosciences
CD40	PE-CF594	Rat IgG2a	3/23	562847	BD Biosciences
CD44	APC-Cy7	Rat IgG2b, κ	IM7	560568	BD Biosciences
CD45	AF700	Rat IgG2b, κ	30-F11	25-0211-82	ebioscience
IgM	PE-CF594	Rat IgG2a, κ	R6-60.2	562565	BD Biosciences
IgD	APC-H7	Rat IgG1	11-26c.2a	565348	BD Biosciences
MHC Class II	APC-eFluor780	Rat IgG2b, κ	M5/114.15.2	47-5321-82	ebioscience
CD69	PE-Cy7	Hamster IgG1	H1.2F3	552879	BD Biosciences
CD80	APC	Hamster IgG2, κ	16-10A1	560016	BD Biosciences
CD86	FITC	Rat IgG2a, κ	GL1	561962	BD Biosciences
CD138	APC	Rat IgG2a, κ	281-2	558626	BD Biosciences
CD138	BV421	Rat IgG2a, κ	281-2	563147	BD Biosciences
CXCR4 (CD184)	FITC	Rat IgG2b, κ	2B11/CXCR4	551967	BD Biosciences
FasL (CD178)	APC	Hamster IgG	MFL3	17-5911-82	eBioscience
ICOS (CD278)	PE-Cy7	Rat IgG2b, κ	7E.17G9	25-9942-82	eBioscience
PD-1 (CD279)	BV421	Hamster IgG2, κ	J43	562584	BD Biosciences
PD-L1 (CD274)	BV421	Rat IgG2a, λ	MIH5	564716	BD Biosciences
PD-L2 (CD273)	PerCP-eFluor710	Rat IgG2a, κ	122	46-9972-82	eBioscience
TACI (CD267)	AF647	Rat IgG2a, κ	8F10	558453	BD Biosciences
Tim-1 (CD365)	BV421	Rat IgG2b, κ	RMT1-4	566336	BD Biosciences
			Intracellular		
Foxp3	PE-CF594	Rat IgG2a, κ	FJK-16s	61-5773-82	eBioscience
CTLA-4 (CD152)	PE	Rat IgG1, κ	UC10-4F10-11	553720	BD Biosciences
IFN-γ	BV421	Rat IgG1, κ	XMG1.2	563376	BD Biosciences
IL-10	PE	Rat IgG2b	JES5-16E3	554467	BD Biosciences
IL-10	APC	Rat IgG2b	JES5-16E3	17-7101-82	BD Biosciences
IL-17A	PE	Rat IgG1, κ	TC11-18H19	559502	BD Biosciences
IL-17A	PE-Cy7	Rat IgG2a, κ	eBio17B7	25-7177-82	eBioscience
TNF-α	PE	Rat IgG1	MP6-XT22	554419	BD Biosciences
TNF-α	PerCP-Cy5.5	Rat IgG1	MP6-XT22	560659	BD Biosciences
			Cell viability dye		
FVD	BV510			65-0866-18	eBioscience

Table S2: Flow cytometry panels used in this study

Panel 1	Panel 2	Panel 3	Panel 4	Panel 5	Panel 6	Panel 7	Panel 8
CD5	CD24	Tim-1	PD-L1	PD-1	IFN-γ	CD14	Ly-6G
FVD	FVD	FVD	FVD	FVD	FVD	B220	NOS2
B220	B220	CD138	B220	CD4	B220	F4/80	CD11b
CD1d	CD22.2	CXCR4	CD11b	CTLA-4	CD4	CD11c	CD62L
IL-10	CD23	CD1d	CD86	Foxp3	TNF-α		
CD40	CD1d	IL-10	PD-L2	ICOS	IL-10		
IL-17A	IL-10	IgM	IL-10	CD25	Foxp3		
FasL	IgM	TACI	CD11c	CD45	IL-17A		
CD45	CD21	CD45	CD69	CD8	CD25		
	CD138	CD44	CD80		CD45		
	CD45		CD45		CD8		
	IgD		MHC II				

Table S3: Score, which was used to define maternal well-being 24 h after LPS injection at gd16

rating	measures	observation
Score 0	no burden, animals healthy	Shining fur, clear eyes, normal posture, spontaneous motility, normal breathing, no clinical complications
Score 1	low burden, low sickness	matte fur, clear eyes, normal posture, reduced motility, normal breathing, no clinical complications
Score 2	moderate burden, moderate sickness	matte fur, unclean eyes, hunched posture, severe reduced motility, breathing slightly changed, no clinical complications
Score 3	severe burden, severe sickness	ruffled fur, (semi-) closed eyes, massively hunched posture, isolation, lethargy, tachypnoea, breath noises, tension, tremor, animals feel cold to the touch
Score 4	death	
